# Broad and Narrow Transdiagnostic Risk Factors in Eating Disorders: A Preliminary Study on an Italian Clinical Sample

**DOI:** 10.3390/ijerph19116886

**Published:** 2022-06-04

**Authors:** Sara Iannattone, Silvia Cerea, Eleonora Carraro, Marta Ghisi, Gioia Bottesi

**Affiliations:** 1 Department of General Psychology, University of Padova, 35131 Padova, Italy; silvia.cerea@unipd.it (S.C.); eleonora.carraro@studenti.unipd.it (E.C.); marta.ghisi@unipd.it (M.G.); gioia.bottesi@unipd.it (G.B.); 2 Hospital Psychology Unit, University-Hospital of Padova, 35128 Padova, Italy

**Keywords:** eating disorders, intolerance of uncertainty, emotion dysregulation, transdiagnostic factors, patients, body image

## Abstract

Eating disorders are multifaceted psychopathologies and the transdiagnostic approach is currently considered a useful framework to understand their complexity. This preliminary study aimed to investigate both broad (i.e., intolerance of uncertainty and emotion dysregulation) and narrow (i.e., extreme body dissatisfaction) transdiagnostic risk factors underlying eating disorders. 50 Italian female patients seeking treatment for an eating disorder were involved (*M*_age_ = 31.6 years ± 12.8, 18–65). They completed self-report measures assessing emotion regulation difficulties, intolerance of uncertainty, extreme body dissatisfaction, general psychological distress, and eating disorder symptomatology. To explore whether the abovementioned transdiagnostic factors predicted patients’ psychological distress and eating disorder symptoms, two linear regressions were performed. Emotion dysregulation emerged as the only significant predictor of distress, while extreme body dissatisfaction was the only significant predictor of overall eating disorder symptomatology. Then, to analyze the differences between patients with anorexia nervosa and bulimia nervosa in intolerance of uncertainty and emotion regulation problems, *t*-tests were conducted. The two groups differed significantly in intolerance of uncertainty levels only, with higher scores obtained by patients with anorexia nervosa. Overall, our findings suggest that emotion dysregulation and extreme body dissatisfaction may be relevant constructs in eating disorders in general, while intolerance of uncertainty may be more involved in restrictive eating disorders. The clinical implications of such results are discussed.

## 1. Introduction

Eating Disorders (EDs) are severe and debilitating mental illnesses characterized by dysfunctional eating and eating-related behaviors (e.g., starvation and self-induced vomiting), and excessive concerns over shape, weight, and body image [1]. EDs constitute a major public health concern since they are associated with negative life outcomes, in particular social, emotional, and cognitive impairment (e.g., [2,3]), suicidality (e.g., [4,5]), and high mortality rates due to acute and chronic medical complications (e.g., [6,7]). This scenario is further worsened by the low sustained recovery rates and high relapse rates characteristic of eating pathologies [8]; in fact, a significant proportion of patients do not benefit from treatment interventions or discharge prematurely, thus developing a long-standing illness [9,10].

A possible reason behind the impaired quality of life and treatment resistance of patients with EDs is that they often meet the criteria for other mental disorders [11,12]. Specifically, comorbidity in EDs was found to increase symptoms severity [13] and the risk of suicidal behaviors [14], and undermine treatment outcomes [15,16]. One of the most prevalent psychological disorders in patients with EDs is Body Dysmorphic Disorder (BDD) [17,18,19,20]. BDD is characterized by concerns with one or more perceived defects in physical appearance that are not observable or appear very slight to others [1]. The preoccupation is time-consuming and causes significant distress and/or impairment in the individual’s functioning [21]. BDD is also characterized by time-consuming repetitive behaviors (e.g., camouflaging, mirror checking, and reassurance seeking), avoidance (e.g., of social situations, mirrors), and high psychiatric comorbidity [22]. Extreme body dissatisfaction is at the core of both EDs and BDD, together with other clinical features, such as intrusive thoughts about physical appearance and overemphasis on physical appearance to determine self-worth (e.g., [23,24]). Comorbid BDD in patients with EDs seems to confer additional severity to clinical symptomatology and, if not recognized and treated promptly, can increase the risk of relapse (e.g., [17,19,25]).

Bearing all this in mind, it is crucial to expand our understanding of the complex patterns of comorbidity among EDs, implement effective treatment programs, and improve the psychological well-being of patients. To this end, the literature highly recommends approaching eating pathology from a transdiagnostic perspective, which is currently considered the most realistic representation of psychological disorders since it reflects the complexity, dimensionality, and comorbidity commonly found in clinical practice [26]. In particular, the transdiagnostic approach involves the investigation of the common and core dimensions underlying a broad range of diagnostic presentations (e.g., [27,28]); in contrast, the traditional approach to EDs is disorder-specific as it focuses mainly on symptoms strictly related to eating pathology and pays less attention to comorbidity (e.g., [29,30]). The transdiagnostic model of EDs represents a dimensional approach that cuts across traditional categorical diagnoses and goes beyond them by considering the processes that are relevant to both eating pathology and other psychological disorders [26,31]; therefore, the study of transdiagnostic vulnerability factors in EDs is particularly important since it may enable interventions to target such factors, thus treating comorbid disorders simultaneously and enhancing treatment effectiveness [32]. In this regard, two mechanisms worthy of further research, especially in the EDs field, are emotion dysregulation and Intolerance of Uncertainty (IU).

### 1.1. Emotion Dysregulation

According to Gratz and Roemer [33], emotion regulation (ER) is a multifaceted construct that includes the following dimensions: (a) awareness and understanding of emotions, (b) acceptance of emotions, (c) ability to act in accordance with desired goals and regulate impulsive behaviors when experiencing negative emotions, (d) ability to use flexible and situationally appropriate ER strategies to modulate the intensity and/or duration of emotional responses. In case of the absence of any or all of these abilities, people experience emotion dysregulation, which is a recognized transdiagnostic vulnerability factor involved both in internalizing and externalizing psychopathology [34,35,36]. In addition, changes in ER during different interventions were found to be associated with an improvement in the symptomatology of several psychological disorders, including EDs (e.g., [37]).

The role of emotion dysregulation as a crucial factor in the etiology and maintenance of EDs is widely supported in the literature (e.g., [35,38]). In fact, extensive research pointed out that eating pathology was related to high emotion dysregulation levels and poor adaptive ER strategies in both clinical and non-clinical adult samples [39,40,41]. From a qualitative standpoint, a recent meta-synthesis showed that patients with EDs have difficulty managing their emotions, so they use ED behaviors to deal with and control negative emotions [42]. This latter aspect provided further support to previous empirical studies, which highlighted the function of ED symptoms and related behaviors as strategies for regulating emotions and coping with the negative arousal generated by emotions themselves [35,43,44].

The research conducted so far has found higher emotion dysregulation levels in patients with EDs compared to healthy controls (e.g., [45,46]). Nevertheless, data are still inconclusive as regards the differences between Anorexia Nervosa (AN) and Bulimia Nervosa (BN) in terms of their relationships with specific ER dimensions. For example, considering ER as assessed by the *Difficulties in Emotion Regulation Scale* (DERS; [33]), several works did not highlight significant differences between AN and BN on any of the scales (e.g., [46,47,48,49]). Other studies, instead, found significantly higher scores in patients with BN on most scales [45,50,51,52,53]; a notable exception was emotional awareness, which was found not to differ between patients with AN and BN in all of the above studies. Finally, recent meta-analyses and systematic reviews, which included studies conducted using different ER measures, concluded that global ER problems may be a transdiagnostic trait throughout the ED spectrum, but specific ER difficulties may distinguish between AN and BN [39,54,55]; hence the importance of further investigating ER and its dimensions in the ED population.

### 1.2. Intolerance of Uncertainty

IU is defined as “the tendency to be bothered or upset by the (as yet) unknown elements of a situation, whether the possible outcome is negative or not“ [56] (p. 6). People with high IU levels are prone to difficulties in tolerating and regulating the negative emotions generated by uncertainty and tend to react negatively on an emotional, cognitive, and behavioral level to uncertain circumstances [56,57]. IU was originally studied within the framework of anxiety disorders as a vulnerability factor for worry, the core feature of Generalized Anxiety Disorder (GAD) [58,59]. Nevertheless, current evidence considers IU a transdiagnostic risk factor spanning a wide array of psychological disorders (e.g., [60,61]). Additionally, more recent conceptualizations support the role of IU as a transdiagnostic and trans-therapy change process [62]; to be specific, some works showed that changes in IU were associated with changes in diagnostic outcomes during psychotherapy for various disorders, and interventions focusing on IU when treating different psychopathologies proved effective (e.g., [63,64]).

With particular reference to EDs, a growing body of empirical and theoretical research has shown higher levels of worry and IU in adults with ED symptoms compared to healthy controls in both clinical and non-clinical samples [65,66,67,68,69]. Furthermore, elevated IU was found to be related to more severe ED symptomatology [70]. Therefore, the current literature seems to agree on the important role that IU plays in the development and maintenance of ED attitudes and behaviors [71,72]. Specifically, some authors suggest that individuals with ED symptoms may be generally high in IU, therefore exposure to uncertain events increases their worry; this, in turn, heightens negative affectivity and encourages the use of ED behaviors to prevent further uncertainty and the resulting negative emotional states (e.g., [60,73]). As stated above, patients with EDs also have difficulty regulating their emotions; therefore, it is reasonable to assume that emotion dysregulation plays a role in the path from IU to ED behaviors and symptoms. Nevertheless, the relationship between IU and ER has not been explored in ED samples yet, although the results of preliminary studies on different psychological disorders (e.g., GAD, substance use disorders, borderline personality disorder) suggest that these two constructs may be somehow associated [74,75,76]. For example, the inability to control behaviors when experiencing emotional distress—which is a facet of emotion dysregulation—was found to mediate the path from IU to negative affect in individuals with substance use disorders [75].

A wealth of research has investigated IU in the ED population, but contrasting results emerged when considering AN and BN separately. Studies among patients with AN showed elevated levels of IU (e.g., [66,77]) and significant correlations between IU and drive for thinness [65], thus suggesting that IU may be an important mechanism underlying anxiety and anxiety-related behaviors in AN. Moreover, IU was hypothesized to contribute to the occurrence and maintenance of rigid and obsessive-compulsive traits in patients with restrictive ED [78]. Strong IU in individuals with AN was also supported by qualitative studies: adult and adolescent patients reported feeling overwhelmed when facing uncertainty, which is viewed as a ‘bad’ experience causing distress, worry, and loss of control, and should be avoided [78,79,80]. Furthermore, personal accounts of patients revealed that AN behaviors are strategies serving the purpose of security and control in uncertain circumstances, as they reduce the perceived threat related to uncertainty and help cope with difficult feelings [78,79]. On the other hand, the findings on BN are more limited. Most studies found higher IU levels in patients with AN compared to those with BN (e.g., [79]), and no significant correlation between IU and binge eating behaviors [81,82,83], thus concluding that IU may be more strongly associated with restrictive ED features. Conversely, other works showed similar IU levels in patients with AN and BN, and higher IU levels in individuals with BN compared to healthy controls [65,71].

### 1.3. Aims of the Study

Although research on IU and ER in EDs has gathered considerable momentum in recent years, a great deal is still unknown on this topic. However, an in-depth study of such constructs may be clinically relevant given that the literature recommends taking a transdiagnostic stance to understand the complexity of eating pathology (e.g., [28]), since this enables to improve EDs psychological treatment. This preliminary study sought to build upon and extend past work by considering both broad (i.e., IU and emotion dysregulation) and narrow (i.e., extreme body dissatisfaction) transdiagnostic risk factors implicated in EDs. In fact, the extant research has evaluated the individual contribution of such factors to disordered eating (e.g., [47,82,84]), but no work has considered them jointly so far. Thus, we involved a mixed diagnostic sample of Italian patients with EDs with the twofold aim of:

(a) Exploring the predictive role of IU, emotion dysregulation, and extreme body dissatisfaction on psychological distress (i.e., depression, anxiety, and stress) and ED symptomatology. To our knowledge, this is the first study on EDs that includes all the above transdiagnostic factors as predictors in the same model. Considering each factor individually can be useful to pinpoint the characteristics associated with EDs, but it may be insufficiently explanatory of the relevance of constructs over and above other established ones; hence the importance of evaluating these three transdiagnostic factors together to ascertain the statistical significance of the effect of each factor over and above the effects of one another.

Furthermore, overall distress in patients with EDs is a hitherto neglected aspect, although eating pathology was found to frequently co-occur with anxiety and depression; in particular, these often precede the onset of ED behaviors, negatively affect the course of the disease, and increase treatment complexity (e.g., [85,86]). Consequently, identifying the predictors of general psychological distress can be particularly useful in preventing the development of anxious-depressive symptomatology that, in turn, can pose a risk for the onset of EDs. However, in light of the exploratory nature of such an investigation, we did not formulate any specific hypothesis.

(b) Deepening the differences between patients with AN and BN in terms of IU and specific ER difficulties given that contrasting results emerged in previous studies. We hypothesized to find higher IU levels in patients with AN compared to those with BN (e.g., [79,83]). Pertaining to ER dimensions, we expected no significant differences between the two groups in emotional awareness (e.g., [46,50]); conversely, given the above-mentioned contradictory results, no specific hypotheses were formulated for the other ER dimensions.

## 2. Materials and Methods

### 2.1. Participants

The sample as a whole was made up of 50 Italian female patients seeking treatment for an ED; specifically, 29 (58%) were diagnosed with AN, 13 (26%) with BN, 3 (6%) with Binge Eating Disorder (BED), and 5 (10%) with Other Specified Feeding or Eating Disorders (OSFED). Diagnoses were established by the clinical staff (i.e., clinical psychologists and psychiatrists) using the Structured Clinical Interview for DSM-5 Disorders—Clinical Version [87].

The patients’ mean age was 31.6 years (*SD* = 12.8, *range* = [18,19,20,21,22,23,24,25,26,27,28,29,30,31,32,33,34,35,36,37,38,39,40,41,42,43,44,45,46,47,48,49,50,51,52,53,54,55,56,57,58,59,60,61,62,63,64,65]), and their mean years of education were 13.4 (*SD* = 2.84, *range* = [8,9,10,11,12,13,14,15,16,17,18,19]). As concerns marital status, 60% were single, 28% were in a relationship, 6% were married/in a domestic partnership, and 6% were divorced/separated. Pertaining to occupation, 38.8% of those who responded (*n* = 49) were students, 18.4% unemployed, 14.3% full-time employed, 6.1% occasionally employed, 4.1% part-time employed, 4.1% housewives, 4% unable to work due to disability, 2% retired, and 8.2% other. With specific regard to clinical variables, 40% and 28% of patients were diagnosed with, respectively, one and two comorbid psychological disorders. Finally, among those who responded (*N* = 49 and *N* = 48, respectively), 87.8% were medicated and 79.2% had been hospitalized at least once.

Table 1 presents the sociodemographic and clinical characteristics of the four subgroups of patients (i.e., AN, BN, BED, and OSFED).

### 2.2. Procedure

The patients were recruited in public and private settings specialized in the psychological treatment of EDs located in northern Italy. All patients satisfied the following inclusion criteria: age > 18 and presence of an ED diagnosis; along the same line, non-suitable patients were considered those younger than 18 years, who did not meet the criteria for a full-blown ED, and with a diagnosis of psychotic disorders, major neurocognitive disorder, or intellectual disability.

First, participants were informed of the study aims and were made aware of the voluntary nature of their participation and their right to withdraw from the study at any time. Then, they gave their written informed consent to enter the study. No incentives or rewards were offered for participating.

Participants completed a data collection sheet, containing sociodemographic information, and a battery of self-report questionnaires (see list below); the sequence of measures was rotated to control for order effects. The time taken to complete the survey was approximately 30 min. The clinical staff provided information about the patient’s ED diagnosis, medications, previous hospitalizations, and comorbid psychological disorders. The collected data were then coded, cleaned, and analyzed by researchers confidentially.

The study was approved by the Ethics Committee for the Psychological Research of the University of Padova and was conducted in full agreement with the Declaration of Helsinki.

### 2.3. Measures

The sociodemographic information sheet contained questions about age, sex, marital status, years of education, occupation, and self-reported height and weight to calculate the Body Mass Index (BMI, kg/m^2^).

The *Difficulties in Emotion Regulation Scale* (DERS; [33]) is a 36-item self-report measure that assesses six specific dimensions of emotion dysregulation: non-acceptance of negative emotions (Nonacceptance), difficulties engaging in goal-directed behaviors when experiencing negative emotions (Goals), belief that is particularly difficult to regulate emotions effectively (Strategies), difficulties in maintaining control when distressed (Impulse), lack of emotional awareness (Awareness), and lack of emotional clarity (Clarity). Items are rated on a 5-point Likert scale (1 = *Almost never (0–10%)*, 5 = *Almost always (91–100%)*). The Italian version of the DERS presented a good internal consistency for the total score (Cronbach’s α = 0.90) and the six subscales (α values ranging from 0.74 to 0.88) [88]; an adequate test-retest reliability was also shown, with intraclass correlation coefficients ranging from 0.49 to 0.78 [89].

The *Intolerance of Uncertainty Scale-Revised* (IUS-R; [90]) is a 12-item self-report questionnaire assessing the dispositional characteristic to find uncertainty upsetting and distressing. Each item is rated on a 5-point Likert scale (1 = *Not at all like me*, 5 = *Entirely like me*). The Italian version of the tool showed an excellent internal consistency (Cronbach’s α *=* 0.90, McDonald’s ω_h_ = 0.90) and a good one-month test-retest reliability (*r* = 0.74) in undergraduate and adult samples [91].

The *Depression Anxiety Stress Scales-21* (DASS-21; [92]) is a 21-item measure evaluating depression, anxiety, and stress symptoms over the previous week on a 5-point Likert scale (0 = *Did not apply to me at all*, 4 = *Applied to me very much, or most of the time*). A total score, as well as three subscale scores (i.e., anxiety, depression, stress), can be computed. However, the findings on the Italian version of the scale suggested that the use of the total score, measuring a “general psychological distress factor”, could be more appropriate than calculating the three scale scores separately [93]. The total score of the Italian version showed excellent internal consistency in both clinical (α = 0.92) and nonclinical (α = 0.90) samples; test-retest reliability in the community sample was also excellent (*r* = 0.74).

The *Questionario sul Dismorfismo Corporeo* (QDC; [94]) is a self-report inventory made up of 40 items assessing extreme body dissatisfaction and BDD symptoms. Respondents are asked to evaluate the extent to which each statement applies to them on a 7-point Likert scale (from 1 = *Strongly disagree* to 7 = *Strongly agree*). The QDC assesses the presence of behaviors associated with extreme body dissatisfaction and BDD, such as repetitive behaviors, mental acts, and avoidance behaviors; it also evaluates the request for cosmetic and surgical procedures and the presence of suicidal thoughts due to appearance concerns. The internal consistency and 1-month test-retest reliability were excellent (α = 0.95 and *r* = 0.91, respectively). The QDC also showed high specificity and sensitivity with a cut-off point of 130, indicating that individuals who obtained a score above 130 should be further assessed because they might present extreme body dissatisfaction/BDD symptoms or may be at risk of developing BDD [94].

The *Eating Disorder Inventory-3* (EDI-3; [95]) is a questionnaire assessing the psychological constructs and behaviors that are clinically relevant in EDs. Each item is rated on a 6-point Likert scale (1 = *Always*, 6 = *Never*). The tool is made up of 91 items organized into 12 primary scales, consisting of 3 ED-specific scales and 9 general psychological scales. The ED-specific scales, namely Drive for Thinness (DT), Bulimia (B), and Body Dissatisfaction (BD), compose the Eating Disorder Risk Composite (EDRC) score; this reflects the overall level of eating concerns and ED symptoms. The Italian version of the EDI-3 demonstrated good internal consistency in both clinical (α = 0.70–0.94) and nonclinical samples (α = 0.55–0.92) [96]. For the purposes of the current study, we considered the EDRC score only.

Table 2 shows the published means and standard deviations of the Italian version of the abovementioned questionnaires, together with their estimated values in the current clinical sample.

### 2.4. Data Analysis

The following analyses were conducted using the Jamovi statistical software [97].

First, descriptive statistics were calculated to define the characteristics of both the whole sample and the subgroups of patients with AN and BN (see Section 2.1).

Subsequently, the overall sample was considered to explore whether IU, emotion dysregulation, and extreme body dissatisfaction significantly predicted psychological distress and global ED symptoms; for this purpose, two hierarchical multiple linear regressions were conducted using the DASS-21 total score and the EDRC scale of the EDI-3 as the outcome variables. Preliminary to such analyses, Pearson’s bivariate correlations were run between the outcome variables (i.e., DASS-21 and EDRC), the scales assessing transdiagnostic risk factors (i.e., IUS-R, DERS, and QDC), and age. For each regression, the variables that resulted significantly correlated with the outcome variable were entered as predictors in two blocks: the control variables were input into the first block, while the transdiagnostic risk factors were entered into the second block (see Section 3.1 and Section 3.2). Conventional statistical significance levels were adopted for hierarchical multiple linear regressions (*p* < 0.05), while *p*-values of the correlations were corrected for multiple comparisons using Bonferroni’s correction (*p* = 0.004).

Finally, independent sample *t*-tests were computed considering the subsample of patients with AN and BN (*N* = 42), in order to investigate the differences between these two groups in terms of IU and ER dimensions. To be specific, the belonging group was the independent variable, while the IUS-R total score and the DERS scale scores were the dependent variables. Cohen’s *d* was used to assess the magnitude of the effects: based on Cohen’s [98] criteria, *d* = 0.2 can be considered a small effect size, *d* = 0.5 a medium effect size, and *d* = 0.8 a large effect size. The level of statistical significance was set at *p* < 0.05 for the IUS-R, while Bonferroni’s correction was applied to the DERS scales (*p* = 0.007).

## 3. Results

### 3.1. Predictors of General Psychological Distress

As a preliminary step, Pearson’s *r* correlations were performed between the DASS-21 and the IUS-R, DERS, QDC, EDRC scale, and age. After correction for multiple comparisons, the DASS-21 resulted significantly associated with the IUS-R (*r* = 0.60, *p* < 0.001), DERS (*r* = 0.76, *p* < 0.001), QDC (*r* = 0.56, *p* < 0.001), and EDRC scale (*r* = 0.42, *p* = 0.004). Therefore, these variables were considered as predictors in the linear regression with the DASS-21 as the dependent variable; specifically, the EDRC scale (i.e., the control variable) was input into the first block, while the IUS-R, DERS, and QDC (i.e., the transdiagnostic risk factors) were entered into the second block. The result of the regression showed that the final model explained 52.8% of the variance in the DASS-21 (Table 3). Specifically, entering the control variables in the first step accounted for 22.7% of the variance in the DASS-21 (Model 1); then, the inclusion of the IUS-R, DERS, and QDC total scores in the second step (Model 2) accounted for an additional 30.1% of explained variance (model comparison: Δ*R*^2^ = 0.328, *F* (3, 37) = 9.50, *p* < 0.001). In the final model, the DERS total score emerged as the only significant predictor (*p* = 0.002), while the EDRC scale was no longer significant (*p* = 0.14).

### 3.2. Predictors of Overall ED Symptoms

When the correlations between the EDRC scale and the DASS-21, IUS-R, DERS, QDC, and age were run, the only ones surviving the Bonferroni’s correction were those with the DASS-21 (*r* = 0.42, *p* = 0.004) and the QDC (*r* = 0.52, *p* < 0.001). Consequently, these variables were input as predictors into the regression model with the EDRC as the outcome variable. In particular, the DASS-21 (i.e., the control variable) was entered into the first block, while the QDC (i.e., the transdiagnostic risk factor) was entered into the second block. The result of the regression pointed out that the model overall explained 29.5% of the variance in the EDRC (Table 4). The inclusion of the DASS-21 in the first block (Model 1) accounted for 22.7% of the variance; subsequently, when the QDC was added in the second block (Model 2), an additional 6.8% of variance was explained by the model (model comparison: Δ*R*^2^ = 0.08, *F* (1, 39) = 4. 87, *p* = 0.03). In the final model, the QDC total score was the only significant predictor (*p* = 0.03), while the DASS-21 was no longer significant (*p* = 0.08).

### 3.3. Differences between Patients with AN and BN in IU and ER Difficulties

Independent sample *t*-tests were conducted considering the subsample of patients with AN and BN.

When the IUS-R was input as the dependent variable, a significant difference between the two groups emerged (*t*_40_ = 2.3, *p* = 0.03), and the effect size was medium-large in magnitude (Cohen’s *d* = 0.77); specifically, patients with AN showed higher IU levels compared to patients with BN (*M*_AN_ = 39.8, *SE* = 2.33; *M*_BN_ = 30.5, *SE* = 3.15).

On the contrary, when the DERS scales were inserted as the dependent variables, no significant results emerged (all *p*s > 0.35), thus highlighting that patients with AN and BN did not differ significantly in terms of both overall emotion dysregulation levels and specific ER difficulties.

## 4. Discussion

The current preliminary study was designed to gain deeper insight into the transdiagnostic vulnerability factors implicated in EDs; although research in this field is flourishing, some issues remain still unsolved or barely investigated. Therefore, a sample of Italian female patients with EDs was involved to examine both broad—i.e., IU and emotion dysregulation—and narrow—i.e., extreme body dissatisfaction—transdiagnostic risk factors thought to underlie eating pathology, thus promoting a more nuanced understanding of such an important topic.

The first specific aim was to explore the predictive role of IU, emotion dysregulation, and extreme body dissatisfaction on the patients’ psychological distress (i.e., anxiety, depression, and stress) and ED symptoms. Such transdiagnostic factors have often been considered individually in relation to EDs (e.g., [47,82,84]), while we are unaware of any published work that has included them in the same model or study.

With regard to general distress, it was found to be highly correlated with all the transdiagnostic factors considered. Nevertheless, when said factors were evaluated concurrently and the effect of overall ED symptomatology was controlled, emotion dysregulation emerged as the only significant predictor of the patients’ general psychological distress, over and above the effects of IU and extreme body dissatisfaction. Consequently, this result would suggest that difficulties in ER may represent the core mechanism underpinning general distress in patients with EDs, while IU and extreme body dissatisfaction may marginally contribute to general distress. To our knowledge, research has mainly investigated emotion dysregulation and psychological distress separately in patients with EDs, while the relationship between these two variables is still unexplored in such a population. However, the following independent lines of evidence could provide support to our preliminary result and lead to a step-by-step understanding of it: (1) the presence of ER difficulties is a well-established mechanism involved in eating pathology (e.g., [35,38]); (2) emotion dysregulation is a transdiagnostic factor found to be associated with anxiety and depressive disorders in different populations (e.g., [37,99]); (3) anxiety and depressive symptoms have been recognized as precipitating and maintenance factors for EDs [85,86]; (4) ED behaviors—such as vomiting, bingeing, and restriction—may represent cognitive avoidance strategies performed to block/escape from negative affect (i.e., anxiety and depression) [100] and/or means to cope with difficult emotional states (e.g., [35,43,44]). In light of our findings and the aforementioned arguments, it is possible to surmise that patients with EDs may be characterized by deficits in ER that may heighten the risk of experiencing general psychological distress—expressed in terms of anxiety, depression, and stress symptoms—in response to emotions perceived as overwhelming and difficult to regulate; general distress, in turn and together with other factors, may contribute to increasing the likelihood of habitual usage of ED behaviors to deal with negative affect and emotions, in the face of an inability to find more adaptive coping strategies. ED behaviors may then be maintained through a negative reinforcement mechanism related to a temporary decrease in negative emotional states and distress (e.g., [35]). However, further research is needed to investigate which other variables could intervene in the path from emotion dysregulation to distress in EDs. In this regard, a relevant high-order construct may be neuroticism; this is a personality trait characterized by the tendency to experience negative affect and difficulties in ER [101] and was found to be involved in eating pathology [102]. Therefore, neuroticism in patients with EDs may underlie emotion dysregulation and, at the same time, facilitate the experience of negative affect as a reaction to unpleasant and uncontrollable emotional states. Future studies are encouraged to better understand the role of neuroticism in the relationship between ER difficulties, psychological distress, and ED attitudes.

Pertaining to overall ED symptomatology, instead, the only transdiagnostic factor found to be a significant predictor was extreme body dissatisfaction, above and beyond the effect of general distress. This evidence appears to indicate that extreme body dissatisfaction may have a strong influence on the development of ED behaviors and symptoms, while IU and emotion dysregulation may play a minor role. Specifically, extreme body dissatisfaction as assessed by the QDC refers to severe dissatisfaction with overall physical appearance, which also includes concerns over body parts not strictly related to weight and shape (henceforth non-weight-related body image concerns, NWRCs) [94]. Consequently, our finding tentatively suggests that, although extreme dissatisfaction with body weight and shape is a well-established mechanism in eating pathology (e.g., [1]), high rates of NWRCs may also represent a relevant risk factor for the development of ED symptoms; in particular, ED behaviors may serve as maladaptive coping strategies performed to alleviate the negative thoughts and feelings experienced about different body areas, both related and unrelated to weight and shape. Moreover, 80.9% of the patients scored above the QDC cut-off score, thus further highlighting that an extreme dissatisfaction with overall physical appearance may be common among people with EDs and may act as a risk and/or maintenance factor for eating pathology. Our result aligns with previous studies showing the clinical relevance of assessing NWRCs in people with EDs (e.g., [17,18,19]). Indeed, NWRCs can be conceptualized as a subclinical manifestation of BDD, so early recognition and treatment of NWRCs in patients with EDs may be fundamental to preventing the development of BDD symptoms, which would increase the severity of EDs clinical symptomatology, and enhance treatment effectiveness (e.g., [17,19,23]). Given the potentially relevant role of NWRCs in the development of ED symptoms, future research in this direction is warranted.

Our second aim was to investigate whether patients with AN and BN differed significantly in terms of IU and ER difficulties; in fact, several works have been conducted on these aspects, but contrasting evidence emerged.

As far as IU is concerned, results confirmed our hypothesis given that patients with AN obtained significantly higher IUS-R scores compared to patients with BN. This finding adds to a growing literature showing the clinical relevance of IU in restrictive ED (e.g., [60,72,78]). In particular, patients with AN may present a difficulty tolerating uncertainty since a core feature of the disorder is the need for certainty and structure that leads to the implementation of rigid and control behaviors; conversely, a key diagnostic characteristic of BN is loss of control [83,103]. Therefore, food restriction and ritualistic/obsessive behaviors in AN may develop as means to dampen intolerable feelings of uncertainty and doubt, and increase a sense of control (e.g., [72,77]). Taken together, our result and previous evidence seem to highlight that patients with AN may rely heavily on restrictive behaviors to face uncertainty, and this may also partly explain why they frequently develop beliefs about the positive function of AN in their lives, thus making such behaviors very difficult to extinguish (e.g., [80]). Future studies should try to clarify whether IU is a transdiagnostic factor across the whole ED spectrum or is a peculiar characteristic of restrictive ED.

Finally, with respect to ER, our study showed no significant differences between patients with AN and BN in either overall emotion dysregulation or specific ER strategies. Such a result provides additional support to previous empirical works pointing out that emotion dysregulation, as assessed by the DERS, may not be diagnosis-specific, but may represent a transdiagnostic risk and/or maintenance factor in both AN and BN (e.g., [46,47,48,49]). In particular, as also stated above, individuals with AN and BN may display limited access to adaptive ER strategies; therefore, ED behaviors may be considered as attempts to cope with negative emotional states by providing short-term comfort or distraction (e.g., [35,43,44]). Nonetheless, despite being both marked by emotion dysregulation, AN and BN present different core diagnostic features, which may explain the differences in the particular type of dysfunctional strategy used to cope with negative emotions; specifically, in BN negative emotions may be regulated mainly by pathological eating behaviors, such as binging and vomiting, while in AN by restrictive behaviors, such as excessive exercise and dieting [48]. Although the general pattern of findings yields preliminary support to the fact that global ER difficulties may characterize both AN and BN, it is also possible that such disorders are featured by an impairment in specific ER domains that may be different from those impaired in other psychopathologies. Therefore, future research should resume investigating specific ER difficulties in eating pathology to enable the adaptation of existing and the development of new interventions tailored to patients with EDs.

Despite the intriguing findings that emerged from this study, several limitations should be considered.

The first shortcomings relate to the characteristics of the sample. In fact, the whole sample size was relatively small and only female patients were involved, thus reducing power and raising questions about the representativeness of treatment-seeking patients with EDs. In addition, all patients were receiving treatment, and most of them were medicated, thus limiting the generalizability of our conclusions to patients with EDs in the community and not asking for treatment. Then, no information was collected about treatment duration. However, the patients in the present study differed regarding the duration of psychological/psychiatric treatment they have been receiving; therefore, it cannot be ruled out that the patients’ treatment status may have somehow influenced the results. Moreover, with a limited and unequal subgroup size, caution must be applied when generalizing our results to the whole ED spectrum. In particular, the excessively small number of participants in the BED and OSFED groups did not enable us to compare all the four subgroups of patients as regards IU and ER strategies. Therefore, there is a need for further studies involving a larger and more balanced sample of patients with different ED diagnoses to clarify the role of IU, emotion dysregulation, and extreme body dissatisfaction in eating pathology in general; with a view to achieving greater completeness, such studies should also differentiate between AN restricting type and binge/purge type. Moreover, future works should investigate the predictive role of each ER dimension on general distress and ED symptomatology; due to our limited sample size, we only considered the DERS total score in regression models, but it would be interesting to examine whether specific ER problems can contribute to the development of psychological distress and ED symptoms. Finally, clinical and non-clinical control groups are missing; hence, we cannot exclude that the observed associations are relevant to different populations, rather than specific to patients with EDs.

Subsequently, the cross-sectional design did not enable one to establish the temporal relationship between the variables considered, most of which entertain a bidirectional association. Longitudinal studies should thereby be undertaken to disentangle reciprocal influences.

Lastly, we administered self-report tools only; future investigations should also consider observable behavioral outcomes and employ different methods and measures (e.g., ecological momentary assessment, observational tools, implicit measures).

## 5. Conclusions and Clinical Implications

Despite being preliminary and characterized by the abovementioned limitations, the present study represents the first attempt to examine concurrently three of the most relevant transdiagnostic risk factors in EDs—namely IU, emotion dysregulation, and extreme body dissatisfaction—in a clinical sample. In our opinion, the observed results may have some valuable clinical implications.

First, emotion dysregulation emerged as the only significant predictor of psychological distress in patients with EDs, thus pointing out the importance of considering ER problems as a target in interventions for eating pathology. In particular, EDs programs that include a module directly targeting dysfunctional ER strategies and fostering flexible and adaptive ER skills may be beneficial in treating comorbid anxiety, depression, and stress symptoms, while also reducing ED symptomatology. Efforts directed at increasing people’s ability to endure negative emotions without developing distress may also be useful from a preventive standpoint, given that anxiety and depression were found to frequently precede the onset of EDs (e.g., [85,86]). Moreover, since no significant differences were found in ER abilities between AN and BN, it is reasonable to assume that emotion dysregulation is a transdiagnostic feature at least in both disorders and, therefore, may be commonly targeted.

Subsequently, extreme dissatisfaction with the overall physical appearance was found to predict ED symptomatology; therefore, it would seem that both concerns over body weight and shape and NWRCs may constitute risk and/or maintenance factors for EDs. This result may be particularly helpful in clinical practice given that standard EDs programs focus mainly on eating pathology, while body image disturbance-related symptoms are often ascribed secondary importance (e.g., [104]); however, ad hoc interventions focused on reducing body dissatisfaction should be implemented, as they may represent a promising avenue to treat and prevent EDs.

Finally, evidence from this preliminary study suggests that the area of IU may be critical in AN; in fact, patients with AN appear to be particularly sensitive to uncertainty, and IU may foster the onset and maintenance of restrictive behaviors. Therefore, therapeutic strategies designed to equip patients with more adaptive strategies to cope with uncertainty may be a useful addendum to standard treatments of AN. Furthermore, given that IU is a transdiagnostic factor implicated in both anxiety disorders and restrictive ED, the inclusion of IU as a target in interventions may enable one to treat comorbid anxiety and AN symptoms simultaneously.

In conclusion, EDs are complex and multifaceted psychological disorders, and approaching them from a transdiagnostic viewpoint may enhance assessment, prevention, and treatment. Therefore, we hope that our work may represent a starting point for future research investigating the role of IU, emotion dysregulation, and extreme body dissatisfaction in eating pathology.

## Figures and Tables

**Table 1:** Sociodemographic and clinical characteristics of the four subgroups of patients.

	Group			
	AN (*N* = 29)	BN (*N* = 13)	BED (*N* = 3)	OSFED (*N* = 5)
Mean age (*SD*)	28.6 (10.6)	29.3 (10.3)	56.3 (7.77)	40.8 (14.7)
Mean years of education (*SD*)	14.4 (2.41)	13 (2.08)	9.67 (2.89)	11 (3.94)
Mean BMI (*SD*)	16.6 (2.72)	22.9 (8.43)	30.3 (7.32)	27.4 (10.8)
Marital status				
% Single	65.5	61.5	33.3	40
% In a relationship	31	15.4	33.3	40
% Married/domestic relationship	3.50	7.70	33.4	0
% Divorced/separated	0	15.4	0	20
Occupation				
% Students	46.4	46.2	0	0
% Unemployed	17.9	23.1	0	20
% Full-time employed	14.3	7.70	0	40
% Part-time employed	6.90	0	0	0
% Occasionally employed	3.60	0	33.4	20
% Housewife	0	7.70	33.3	0
% Unable to work due to disability	0	15.4	0	0
% Retired	0	0	0	20
% Other	10.7	0	33.3	0
% Previously hospitalized	85.2	92.3	0	60
% Medicated	82.8	91.7	100	100
Number of comorbidities				
% No comorbidities	31.1	23	66.7	40
% One	44.8	46.2	0	20
% Two	24.1	30.8	33.3	40

*Note.* AN = Anorexia Nervosa; BN = Bulimia Nervosa; BED = Binge Eating Disorder; OSFED = Other Specified Feeding or Eating Disorders; *SD* = Standard Deviation; BMI = Body Mass Index.

**Table 2:** Means and standard deviations for the Italian versions of the tools from published works (general population parameters) and the current sample.

	General Italian Population		Current Sample	
	Mean	*SD*	Mean	*SD*
IUS-R	26.7	8.2	36.4	12.8
DERS				
Total	61.8	15.4	106	30.4
Nonacceptance	11.6	4.59	17.4	7.84
Awareness	5.59	2.6	8.72	3.8
Clarity	7.87	2.5	16.1	5.54
Impulse	10.6	4.53	13.9	6.56
Goals	13	4.37	16.7	5.63
Strategies	11.5	3.69	24.4	8.87
DASS-21	12.3	8.3	28.2	14.4
QDC	105.9	37.5	164	44.7
EDRC	20.7	16.9	51.7	24.4

*Note.* IUS-R = Intolerance of Uncertainty Scale—Revised; DERS = Difficulties in Emotion Regulation Scale; DASS-21 = Depression Anxiety Stress Scale-21; QDC = Questionario sul Dismorfismo Corporeo; EDRC = Eating Disorder Risk Composite; *SD* = Standard Deviation.

**Table 3:** Results of the hierarchical multiple linear regression (outcome variable: DASS-21) considering the whole sample (*N* = 50).

	*B*	*SE*	β	*t*	Adjusted *R*^2^	*F* (df1, df2)
**Model 1**					0.227	13 (1, 40) ***
Intercept	15.4	4.27		3.60 ***		
EDRC	0.27	0.08	0.50	3.61 ***		
**Model 2**					0.528	12.5 (4, 37) ***
Intercept	−11.4	6.33		−1.81		
EDRC	0.10	0.07	0.19	1.51		
DERS Total	0.30	0.09	0.64	3.41 **		
IUS-R Total	−0.14	0.19	−0.13	−0.77		
QDC Total	0.05	0.04	0.16	1.13		

*Note*. The two blocks were also reversed—that is, the DERS, IUS-R, and QDC were input into the first block, while the EDRC was entered into the second block—to test any differences between regression models; however, no differences emerged (the DERS total was the only significant predictor). Model 1: *R* = 0.496, *R*^2^ = 0.246; Model 2: *R* = 0.758, *R*^2^ = 0.574. IUS-R = Intolerance of Uncertainty Scale—Revised; DERS = Difficulties in Emotion Regulation Scale; QDC = Questionario sul Dismorfismo Corporeo; EDRC = Eating Disorder Risk Composite scale; *SE* = Standard Error. *** *p* < 0.001; ** *p* < 0.01.

**Table 4:** Results of the hierarchical multiple linear regression (outcome variable: EDRC scale) considering the whole sample (*N* = 50).

	*B*	*SE*	β	*t*	Adjusted *R*^2^	*F* (df1, df2)
**Model 1**					0.227	13 (1, 40) ***
Intercept	24.9	8.01		3.07 ***		
DASS-21	0.91	0.25	0.50	3.61 ***		
**Model 2**					0.295	9.58 (2, 39) ***
Intercept	2.45	12.8		0.19		
DASS-21	0.53	0.29	0.29	1.82		
QDC Total	0.20	0.09	0.35	2.21 *		

*Note*. The two blocks were also reversed—that is, the QDC was input into the first block, while the DASS-21 was entered into the second block—to to test any differences between regression models; however, no differences emerged (the QDC was the only significant predictor). Model 1: *R* = 0.496, *R*^2^ = 0.246; Model 2: *R* = 0.574, *R*^2^ = 0.329. DASS-21 = Depression Anxiety Stress Scale-21; QDC = Questionario sul Dismorfismo Corporeo; *SE* = Standard Error. *** *p* < 0.001; * *p* < 0.05

## Data Availability

The data presented in this study are available on reasonable request from the corresponding author (S.I.). The data are not publicly available because they report private information about participants.
